# Enteral High-Dose Docosahexaenoic Acid and Neurodevelopment in Extremely Preterm Infants: A Systematic Review and Meta-analysis

**DOI:** 10.1016/j.cdnut.2025.107510

**Published:** 2025-07-24

**Authors:** Emily Shepherd, Naho Ikeda, Thomas R. Sullivan, Isabelle Marc, Mireille Guillot, Andrew J McPhee, Robert A Gibson, Maria Makrides, Jacqueline F Gould

**Affiliations:** 1 Women and Kids Theme, South Australian Health and Medical Research Institute, Adelaide, South Australia, Australia; 2 Adelaide Medical School, University of Adelaide, Adelaide, South Australia, Australia; 3 Department of Pediatrics, Jutendo University Faculty of Medicine, Tokyo, Japan; 4 School of Public Health, University of Adelaide, Adelaide, South Australia, Australia; 5 Department of Pediatrics, Centre Hospitalier Universitaire de Québec-Université Laval, Québec, Québec, Canada; 6 Neonatal Medicine, Women’s and Children’s Hospital, Adelaide, South Australia, Australia; 7 School of Agriculture, Food and Wine, University of Adelaide, Adelaide, South Australia, Australia; 8 School of Psychology, University of Adelaide, Adelaide, South Australia, Australia

**Keywords:** infant, premature, enteral nutrition, DHAs, cognition, systematic review

## Abstract

**Background:**

Enteral high-dose docosahexaenoic acid (DHA) may be required for neurodevelopment, including cognition, of extremely preterm infants. High-level summative evidence is lacking.

**Objectives:**

This study aims to examine associations between enteral high-dose DHA during the neonatal period and neurodevelopment in infants born ≤29 wk of gestation.

**Methods:**

The following databases were searched (from inception to 11 April, 2024): CINAHL, Cochrane Library, Embase, Medline, Scopus, and Web of Science. Eligible randomized controlled trials (RCTs) in infants born ≤29 wk, assessing direct enteral administration ≥ 40 mg/kg/d DHA, or breast milk/formula with DHA ≥ 0.60% total fatty acids, reporting neurodevelopmental outcomes. Two reviewers independently screened articles, extracted data, and assessed quality using the Cochrane Handbook guidance. Data were pooled using fixed or random-effect meta-analyses. The primary outcome was global cognitive scores from a standardized test.

**Results:**

We screened 1978 articles and included 3 high-quality RCTs (2028 infants born ≤29 wk). Enteral high-dose DHA was not associated with overall differences in global cognition scores at a corrected age (CA) of 18–36 mo [3 RCTs, 638 children, mean difference (MD) 0.67; 95% confidence interval (CI): –1.80, 3.15; *P* = 0.59; *I*^2^ = 0%] or CA of 5–7 y (2 RCTs, 852 children; MD: 2.22; 95% CI: –0.14, 4.57; *P* = 0.06; *I*^2^ = 33%); however, benefit was observed in the largest RCT with a direct enteral emulsion (656 children, CA of 5 y, MD 3.45; 95% CI: 0.38, 6.52; *P* = 0.03). Associations with most secondary outcomes were not seen; however, high-dose DHA was associated with reduced mild motor (3 RCTs, CA of 18–36 mo) and cognitive (2 RCTs, CA of 5–7 y) impairment. No negative impacts were observed.

**Conclusions:**

Enteral high-dose DHA in extremely preterm infants was not associated with differences in global cognition scores on meta-analysis; however, higher scores were observed with the use of a direct emulsion. Results support contemporary recommendations.

This trial was registered at PROSPERO as CRD42022382744 (https://www.crd.york.ac.uk/prospero/display_record.php?ID=CRD42022382744).

## Introduction

Despite improvements in survival, infants born preterm remain at high risk for adverse neurodevelopmental outcomes [1]. The likelihood of cognitive, language, motor, and behavioral impairments increases with decreasing gestational age at birth; thus, those born extremely preterm are most vulnerable [2,3].

DHA, an ω-3 long-chain PUFA (LCPUFA), is necessary for early brain development. Placental transfer of DHA and its subsequent fetal accretion in neural tissue is highest during the third trimester of pregnancy [4]. Infants born at <29 wk of gestation are therefore at greatest risk of DHA insufficiency, which is postulated to contribute to compromised neurodevelopment, including cognition [5].

Over the last 2 decades, research on DHA supplementation for preterm infants has shifted focus from the provision of an intake corresponding to that in the breast milk of women consuming a “typically western” diet (∼20 mg/kg/d), to a dose equivalent to the estimated fetal accumulation rate (≥ ∼40–60 mg/kg/d) [6]. Recent narrative reviews and consensus recommendations now also support the likely benefits of high-dose DHA supplementation for preterm infants [7,8]. In its 2022 position paper on enteral nutrition for preterm infants, the European Society for Paediatric Gastroenterology Hepatology and Nutrition suggests that “Providing >50 mg/kg/d of DHA seems sufficient to obtain DHA concentrations like fetal blood in utero,” recommending “a DHA intake range of 30-65 mg/kg/d” in preterm infants with a birthweight <1800 g [7].

To date, however, the randomized controlled trial (RCT) evidence on the long-term neurodevelopmental impacts of high-dose DHA for extremely preterm infants has not been formally synthesized to aid clinical practice and implementation. Thus, we conducted a systematic review and meta-analysis to determine the effects of enteral high-dose DHA supplementation on the neurodevelopment of preterm infants born at <29 wk of gestation.

## Methods

We adhered to the PRISMA reporting guideline [9]. Prior to conduct, the review protocol was registered and published with the PROSPERO (CRD42022382744) [10].

### Eligibility criteria

RCTs or quasi-RCTs, including individually randomized or cluster-RCTs, were eligible. We excluded nonrandomized controlled studies (non-RCTs, cohort studies, case-control studies), cross-sectional studies, case series, and case reports. Studies published as abstract only, along with full-text publications, were considered.

We included RCTs of extremely preterm infants, defined as those born at <29 wk of gestation. If an RCT included eligible and ineligible participants (i.e., infants born at later gestational ages), we planned to only include it where it reported results for eligible participants separately.

We included RCTs of enteral high-dose DHA supplementation, commencing after birth, in the neonatal period, including DHA alone or in conjunction with other LCPUFAs. This was irrespective of the DHA supplementation delivery method, which could include direct enteral supplementation of infants (e.g., via an emulsion), maternal supplementation for enrichment of breast milk, and/or enrichment of preterm formula.

We excluded intravenous DHA supplementation. “High-dose” DHA was considered where the direct enteral supplementation was ≥ 40 mg/kg/d or the target DHA percentage of total fatty acids in breast milk or formula was ≥ 0.60%. We included interventions comparing enteral high-dose DHA with standard care or a control (placebo or lower-dose DHA).

We included RCTs reporting ≥1 of the review outcomes and excluded those that did not report outcomes of interest. *Primary*: global cognitive scores from a standardized test, with an age-standardized score to a mean (***±***SD) of 100 (***±***15). *Secondary*: other measures of neurodevelopment, comprising, as appropriate, scores from a standardized test with an age-standardized score and/or reported impairment, including: other aspects of cognition, language, motor, and behavioral and emotional functioning; along with composite measures of neurodevelopmental impairment.

To encompass all relevant data, we included outcomes as defined and reported by RCT authors, irrespective of assessment methods or tools used. We planned to analyze outcomes separately for infants (< 1 y), toddlers (1–3 y), children in middle (4–8 y), and later childhood/pre-adolescence (9–12 y).

### Data sources and search strategy

Comprehensive searches of the bibliographic databases CINAHL, Cochrane Library, Embase, Medline, Scopus, and Web of Science were undertaken from their inceptions to 4 November, 2024, using combinations of controlled vocabulary (such as Medical Subject Heading terms) and free text words, guided by our “PICO” (Patient, Intervention, Comparison, Outcome) parameters (Supplemental Tables 1–5). No date or language restrictions were applied. We also searched Google Scholar and Google using free text words. The reference lists of eligible references or relevant reviews were checked for additional reports.

Retrieved records were exported to reference manager software EndNote X9 [11], before being uploaded into Covidence for deduplication and screening [12]. After titles and abstracts were screened, we obtained full-text articles for studies that appeared to meet the inclusion criteria. All full-text articles were assessed for inclusion. Each stage of screening was carried out by 2 reviewers (ES and NI, not involved in any of the included publications), and differences were resolved through discussion.

### Data extraction and risk of bias assessment

For included RCTs, data were extracted using a standardized form, including information regarding design, participants, DHA regimen, control, outcomes reported, results relevant to this review, and risk of bias. For all RCTs, extraction was carried out by 2 reviewers (ES and NI), with differences resolved through discussion. Quality appraisal was undertaken by 2 reviewers (ES and NI) utilizing established guidelines provided in the *Cochrane Handbook for Systematic Reviews of Interventions* [13].

### Statistical analysis

Statistical analyses were performed using Review Manager, version 5.4.1 [14]. A 2-sided *P* < 0.05 was considered to be statistically significant. Effect sizes were estimated as risk ratios (RRs) for dichotomous outcomes, and mean differences (MDs) for continuous outcomes, with 95% confidence intervals (CIs). As RCTs mostly presented effect sizes adjusted for randomization stratification variables and/or data clustering (multiple pregnancies) and/or addressed missing data using multiple imputations, the generic inverse variance method was used to pool the effect sizes. We performed a random-effects meta-analysis to combine effect estimates as we anticipated that RCT populations, interventions, and methods would be heterogeneous. However, fixed-effect meta-analysis was used, where only 2 RCTs were included [15].

We planned to explore expected sources of heterogeneity (supplement delivery method): direct enteral emulsion compared with enrichment of breast milk or formula; and gestational age: <27 wk compared with ≥27 wk) through prespecified subgroup analyses for the primary outcome. We planned to conduct sensitivity analyses, restricting primary outcome analyses to RCTs considered at low risk of bias overall, and to assess publication bias through funnel plots.

## Results

### Search results

**FIGURE 1:**
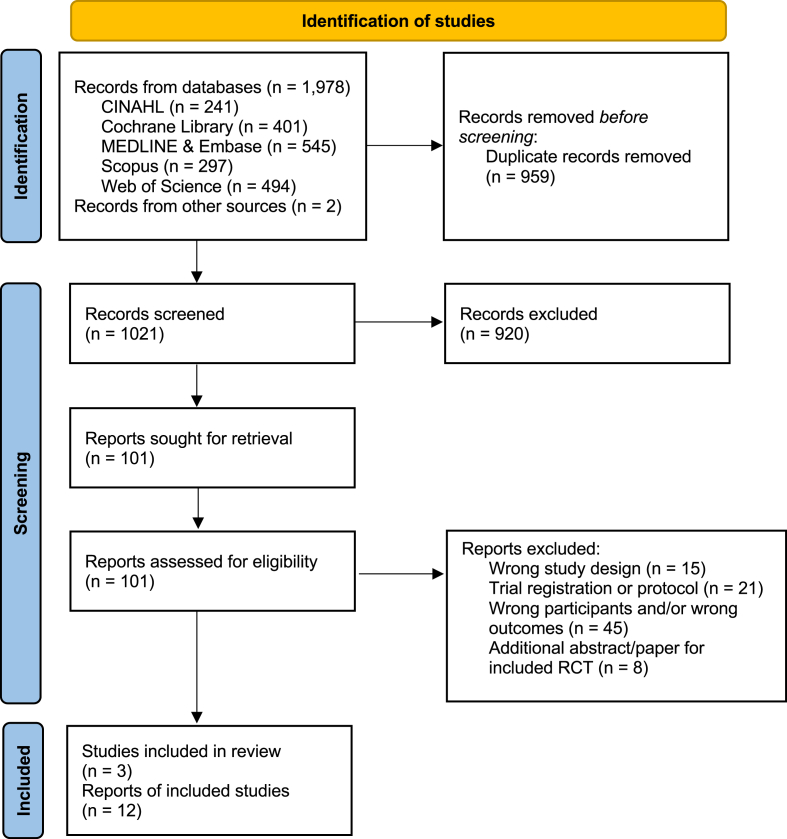
Flow diagram of the selection process.

The searches identified 1978 records; after duplicate removal, there were 1021. Title and abstract screening identified 101 articles for full-text screening, of which we excluded 89 (Supplemental Table 6). We included data from 3 RCTs (information extracted from 12 articles) (Figure 1): the DHA for the Improvement of Neurodevelopmental Outcome in preterm infants trial (DINO) [[16], [17], [18]], the Maternal Omega-3 Supplementation to Reduce Bronchopulmonary Dysplasia in Very Preterm Infants trial (MOBYDIck) [[19], [20], [21]], and the N-3 Fatty Acids for Improvement in Respiratory Outcomes trial (N3RO) [[22], [23], [24], [25], [26], [27]].

### Study characteristics

The 3 RCTs were multicenter, conducted in high-income countries, with recruitment and randomization of 2028 preterm infants born at <29 wk of gestation from 2001 to 2015 [16,19,22]. High-dose DHA delivery method varied across RCTs—in 1 RCT, a direct enteral emulsion was administered to infants [22]; in 2 RCTs, breast milk was enriched via maternal oral supplementation [16,19], in 1 of these RCTs, enriched formula was also available [16]. DHA sources were fish [16,22] and algal oil [19]. In each RCT, the control was a placebo with no additional DHA [16,19,22].

RCTs conducted neurodevelopmental follow-up at corrected age (CA) of 18 mo [18], 18–22 mo [20] or 2–3 y [24], and again at CA of 5 [21,26,27] or 7 y [18]. The review’s primary outcome (global cognitive scores) was assessed in the 3 RCTs at CA of 18–36 mo via the Bayley Scales of Infant and Toddler Development (Bayley) Second (II) [18] or Third (III) [20,24] editions; via the Wechsler Preschool and Primary Scale of Intelligence Fourth Edition (WPPSI-IV) at CA of 5 y [26] and Wechsler Abbreviated Scale of Intelligence (WASI) at CA of 7 y [18]. Secondary review outcomes (including measures of cognition, language and motor development, behavioral, emotional, and executive functioning) were assessed using various methods or tools, including the Bayley-II [18] or III [20,24] at CA of 18–36 mo; and at CA of 5–7 y, the WPPSI-IV [26], WASI [18], Ages and Stages Questionnaire (ASQ) [21], Strengths and Difficulties Questionnaire (SDQ) [18,21,27], and Behavior Rating Inventory of Executive Function (BRIEF) [18,21,27].

Further details of the study and population characteristics, interventions/controls, and neurodevelopmental outcomes measured and reported are provided in Table 1 [16,[18], [19], [20], [21], [22],^24^,^26^,^27^].

**TABLE 1:** Characteristics of included RCTs.

RCT; citation(s) for follow-up	Country (y)	Original RCT population	Follow-up population(s)	Mode of supplementation	DHA group	Control group	Neurodevelopmental outcomes
DINO [16] Gould 2023 [18] (CA of 18 mo and 7 y)	Australia (5 centers) Recruitment: 2001–2005 CA of 18 mo follow-up: 2003–2007 CA of 7 y follow-up: 2008–2013	657 neonates (545 women); <33 wk of GA 227 neonates; <29 wk of GA	<29 wk of GA: CA of 18 mo: 204/208 eligible children (98.1%) CA of 7 y: 196/205 eligible children (95.6%)	Maternal oral supplementation to enrich breast milk, and/or enriched supplementary formula	DHA-rich tuna oil capsules, 6 × 500 mg per day (to achieve ∼ breast milk DHA ∼1% total fatty acids); high-DHA preterm formula (∼1% DHA) if required Commenced within 5 d of receipt of any enteral feeds; continued until infants reached EDD	Placebo capsules (soy oil), 6 × 500 mg/d (no change to breast milk fatty acids); standard preterm infant formula (∼0.35% DHA) if required	CA of 18 mo: Bayley-II: MDI (and <85), PDI (and <85) CA of 7 y: WASI: FSIQ (and <85), VIQ (and <85), PIQ (and <85) SDQ: emotional symptoms score, conduct problems score, hyperactivity/inattention score, peer relationship problems score, prosocial behavior score, impact score, total difficulties score BRIEF: inhibit scale, monitor scale, shift scale, emotional control scale, initiate scale, working memory scale, organization of materials scale, behavioral regulation index, metacognition index; total score: global executive composite
MOBYDIck [19] Guillot 2022 [20] (CA of 18–22 mo) Paquet 2024 [21] (CA of 5 y)	Canada (16 centers) Recruitment: 2015–2018 CA of 18–22 mo follow-up: 2017–2020 CA of 5 y follow-up: 2021–2023	528 neonates (461 mothers); ≥23 to <29 wk of GA	CA of 18–22 mo: 457/528 eligible children (86.6%) CA of 5 y: 132/177 eligible children (74.6%)	Maternal oral supplementation to enrich breast milk	DHA-rich algae oil capsules, providing 1.2 g/d DHA (to achieve breast milk DHA ∼1% fatty acids) Commenced within 72 h of birth until infant reached 36 wk’ PMA	Placebo capsules (50% soy oil, 50% corn oil)	CA of 18–22 mo: Bayley-III: CC (and <85; <70), LC (and <85; <70), MC (and <85; <70) Significant NDI (≥1 of: I CC, LC, MC score <70, CP with GMFCS ≥3, and hearing or visual impairment) Cerebral palsy with GMFCS ≥3 CA of 5 y: ASQ: problem-solving score, personal-social score, fine motor score, gross motor score, communication score SDQ: emotional symptoms score, conduct problems score, hyperactivity score, peer problems score, prosocial behavior score, total difficulties score BRIEF: inhibit scale, shift scale, emotional control scale, working memory scale, plan/organize scale, flexibility index, behavioral regulation index, metacognition index, total score: global executive composite
N3RO [22] Hewawasam 2021 [24] (CA of 2–3 y) Gould 2022–2024 [26,27] (CA of 5 y)	Australia, New Zealand, Singapore (13 centers) Recruitment: 2012–2015 CA of 2–3 y follow-up: 2015–2016 CA of 5 y follow-up: 2018–2021	1273 neonates; <29 wk of GA	CA of 2–3 y follow-up: 56/120 eligible children (46.7%) CA of 5 y follow-up WPPSI-IV: 656 eligible children (100%; multiple imputation used for 176, 26.8%) SDQ and BRIEF: 958 eligible children (100%; multiple imputation used for 227, 23.7%)	Direct enteral supplementation of infants (via nasogastric or orogastric tube)	DHA-rich tuna oil emulsion providing 60 mg DHA per kilogram of body weight per day (administered 3 × per day) Commenced within 3 d after first enteral feeding until 36 wk’ PMA or discharge home, whichever occurred first	Control (soy) emulsion (no additional DHA)	CA of 2–3 y: Bayley-III: CC (and <85), LC (and <85; <70) and MC (and <85) CA of 5 y: WPPSI-IV: FSIQ (and <85; <70), VCC score (and <85), fluid reasoning composite score (and <85), working memory composite score (and <85), processing speed primary index scale score (or WPPSI-III) (and <85), general ability primary index scale score (and <85), and cognitive proficiency primary index scales score (<85) SDQ: emotional symptoms score, conduct problems score, hyperactivity/inattention score, peer relationship problems score, prosocial behavior score, impact score, total difficulties score BRIEF: inhibit scale, shift scale, emotional control scale, working memory scale, plan/organize scale, inhibitory self-control index, flexibility index, emergent metacognition index, total score: global executive composite Neurodevelopmental diagnoses: attention-deficit hyperactivity disorder or attention-deficit disorder, autism spectrum disorder, other behavioral disorder, cerebral palsy, intellectual disability

Abbreviations: ASQ, Ages and Stages Questionnaire; BRIEF, Behavior Rating Inventory of Executive Function; Bayley-II, Bayley Scales of Infant and Toddler Development Second Edition; Bayley-III, Bayley Scales of Infant and Toddler Development Third Edition; CA, corrected age; CC, cognitive composite; CP, cerebral palsy; DINO, DHA for the Improvement of Neurodevelopmental Outcome in preterm infants; GA, gestational age; EDD, estimated date of delivery; FSIQ, full-scale intelligence quotient; GMFCS, Gross Motor Function Classification System; LC, language composite; MC, motor composite; MDI, mental development index; MOBYDIck, Maternal Omega-3 Supplementation to Reduce Bronchopulmonary Dysplasia in Very Preterm Infants; NDI, neurodevelopmental impairment; N3RO, N-3 Fatty Acids for Improvement in Respiratory Outcomes; PDI, psychomotor development index; PMA, postmenstrual age; PIQ, performance intelligence quotient; RCT, randomized controlled trial; SDQ, Strengths and Difficulties Questionnaire; WASI, Wechsler Abbreviated Scale of Intelligence; WPPSI-IV, Wechsler Preschool and Primary Scale of Intelligence Fourth Edition; VCC, verbal comprehension composite; VIQ, verbal intelligence quotient.

The RCTs were assessed as having low risk of bias [16,19,22]. However, there were concerns regarding attrition in 2 RCTs, at CA of 18 mo [24], and CA of 5 y [21] (Supplemental Table 7 and Supplemental Figure 1).

### Quantitative data synthesis of global cognitive scores and other outcomes

#### Outcomes at < 1 y

Not reported by included RCTs.

#### Outcomes at 1–3 y

**FIGURE 2:**
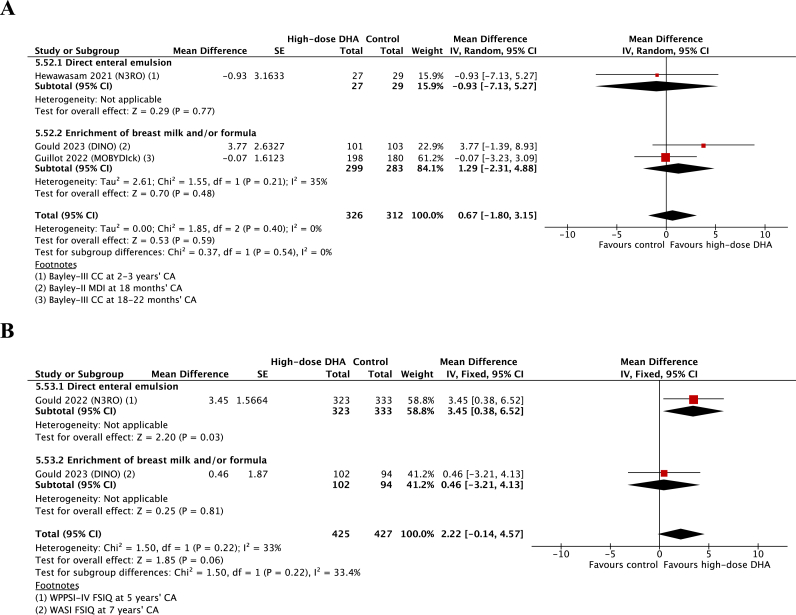
Meta-analysis comparing high-dose DHA supplementation with a control on global cognition scores at 1–3 y (A) and 4–8 y (B). Bayley-II, Bayley Scales of Infant and Toddler Development Second Edition; Bayley-III, Bayley Scales of Infant and Toddler Development Third Edition; CA, corrected age; CC, cognitive composite; CI, confidence interval; DINO, DHA for the Improvement of Neurodevelopmental Outcome in preterm infants; FSIQ, full-scale intelligence quotient; IV, inverse variance; MDI, mental development index; MOBYDIck, Maternal Omega-3 Supplementation to Reduce Bronchopulmonary Dysplasia in Very Preterm Infants; N3RO, N-3 Fatty Acids for Improvement in Respiratory Outcomes; WASI, Wechsler Abbreviated Scale of Intelligence; WPPSI-IV, Wechsler Preschool and Primary Scale of Intelligence Fourth Edition.

Primary outcome: enteral high-dose DHA supplementation was not associated with a difference in global cognition scores [Bayley-II mental development index (MDI) or Bayley-III cognitive composite (CC)] at CA of 18–36 mo (3 RCTs, 638 children, MD 0.67; 95% CI: −1.80, 3.15; *P* = 0.59; *I*^2^ = 0%) (Figure 2). Subgroup analysis indicated no clear differential treatment effect based on supplement delivery method (*P* = 0.54) (Figure 2). Subgroup analysis based on gestational age was not possible, with data stratified by gestation in only 1 RCT [20].

Secondary outcomes (Table 2 [18,20,24] and Supplemental Figure 2): Cognition*:* there was no association between high-dose DHA and mild (Bayley-II MDI or Bayley-III CC < 85) (3 RCTs, 638 children) or moderate/severe cognitive impairment (Bayley-III CC < 70) (1 RCT, 378 children) at CA of 18–22 mo.

**TABLE 2:** Results for neurodevelopmental outcomes at 1–3 y.

Outcome	RCTs	*N*	Methods (*I*^2^)	MD/RR (95% CI)
Primary outcome				
Global cognitive scores: Bayley-II MDI or Bayley-III CS	3 [18,20,24]	638	MD, R (0%)	0.67 (–1.80, 3.15)
Secondary outcomes				
Cognition				
Mild impairment: Bayley-II MDI or Bayley-III CC <85	3 [18,20,24]	638	RR, R (12%)	0.77 (0.51, 1.14)
Moderate/severe impairment: Bayley-III CC <70	1 [20]	378	RR, F (NA)	1.73 (0.65, 4.60)
Language				
Bayley-III LC	2 [20,24]	421	MD, F (0%)	2.43 (–0.77, 5.62)
Mild impairment: Bayley-III LC <85	2 [20,24]	421	RR, F (0%)	0.81 (0.61, 1.06)
Moderate/severe impairment: Bayley-III LC <70	2 [20,24]	422	RR, F (0%)	0.81 (0.48, 1.35)
Motor				
Bayley-II PDI or Bayley-III MC	3 [18,20,24]	615	MD, R (0%)	1.00 (–1.33, 3.33)
Mild impairment: Bayley-II PDI or Bayley-III MC <85	3 [18,20,24]	615	RR, R (0%)	0.79 (0.64, 0.98)^1^
Moderate/severe impairment: Bayley-III MC <70	1 [20]	356	RR, F (NA)	1.41 (0.65, 3.06)
Cerebral palsy (GMFCS ≥3)	1 [20]	403	RR, F (NA)	1.91 (0.17, 21.46)
Composite measures				
Significant neurodevelopmental impairment	1 [20]	352	RR, F (NA)	0.91 (0.57, 1.45)

Test for heterogeneity represented by *I*^2^ statistic.

Abbreviations: Bayley, Bayley Scales of Infant and Toddler Development; CC, cognitive composite; CI, confidence interval; F, fixed effects; GMFCS, Gross Motor Function Classification System; LC, language composite; MC, motor composite; MD, mean difference; MDI; mental development index; *N*, number of participants; NA, not applicable; PDI; psychomotor development index; R, random effects; RCTs, randomized controlled trials; RR, risk ratio.

1 Statistically significant effect estimates.

*Language:* high-dose DHA was not associated with differences in language development scores [Bayley-III language composite (LC)], or mild (Bayley-III LC < 85) or moderate/severe impairment (Bayley-III LC < 70) (all in 2 RCTs, 421 children) at CA of 18–22 mo.

*Motor:* there were no associations between high-dose DHA and motor development scores [Bayley-II psychomotor development index (PDI), or Bayley-III motor composite (MC)] (3 RCTs, 615 children), moderate/severe impairment (Bayley-III MC < 70) (1 RCT, 356 children), or cerebral palsy (1 RCT, 403 children) at CA of 18–22 mo. However, high-dose DHA was associated with a reduction in mild motor impairment (Bayley-II PDI or Bayley-III MC < 85) at CA of 18–22 mo (3 RCTs, 615 children; RR 0.79; 95% CI: 0.64, 0.98; *P* = 0.03; *I*^2^ = 0%).

*Behavioral and emotional functioning:* not reported by included RCTs.

*Composite measure of impairment:* high-dose DHA was not associated with a difference in significant neurodevelopmental impairment at CA of 18–22 mo (1 RCT, 352 children).

#### Outcomes at 4–8 y

Primary outcome: enteral high-dose DHA supplementation was not associated with a difference in global cognitive scores [WASI or WPPSI-IV full-scale intelligence quotient (FSIQ)] at CA of 5–7 y (2 RCTs, 852 children, MD 2.22; 95% CI: −0.14, 4.57; *P* = 0.06; *I*^2^ = 33%) (Figure 2). Although subgroup analysis indicated no clear differential treatment effect based on supplement delivery method (*P* = 0.22) (Figure 2), in the 1 RCT that used a direct enteral emulsion, a benefit was observed (WPPSI-IV FSIQ at CA of 5 y; 656 children, MD 3.45; 95% CI: 0.38, 6.52; *P* = 0.03).

Secondary outcomes (Table 3 [18,21,26,27] and Supplemental Figure 3): *Cognition:* there were largely no associations between high-dose DHA and measures of cognitive development, including verbal comprehension/intelligence [WPPSI-IV verbal comprehension composite (VCC) or WASI verbal intelligence quotient (VIQ)] or impaired comprehension/intelligence (WPPSI-IV VCC or WASI VIQ < 85) at CA of 5–7 y (2 RCTs, 852 children); other indexes of the WPPSI-IV at CA of 5 y (1 RCT, 656 children), WASI at CA of 7 y (1 RCT, 196 children); and problem-solving scores (ASQ) at CA of 5 y (1 RCT, 132 children). However, high-dose DHA was associated with a higher general ability primary index scale score (WPPSI-IV) (MD 3.36; 95% CI: 0.30, 6.42; *P* = 0.03) and reduction in scores < 85 on this scale (RR 0.75; 95% CI: 0.56, 1.00; *P* = 0.05) at CA of 5 y (1 RCT, 656 children). Furthermore, there was a reduction in mild cognitive impairment (WPPSI-IV or WASI FSIQ < 85) with high-dose DHA compared with control at CA of 5–7 y (2 RCTs, 852 children; RR 0.79; 95% CI: 0.62, 1.00; *P* = 0.05; *I*^2^ = 0%).

**TABLE 3:** Results for neurodevelopmental outcomes at 4–8 y.

Outcome	RCTs	*N*	Methods (*I*^2^)	MD/RR (95% CI)
Primary outcome				
Global cognitive scores: WASI FSIQ or WPPSI-IV FSIQ	2 [18,26]	852	MD, F (33%)	2.22 (–0.14, 4.57)
Secondary outcomes				
Cognition				
Verbal scores: WPPSI-IV VCC or WASI VIQ	2 [18,26]	852	MD, F (31%)	2.47 (–0.13, 5.08)
Verbal scores: WPPSI-IV VCC < 85 or WASI VIQ < 85	2 [18,26]	852	RR, F (31%)	0.79 (0.60, 1.03)
WPPSI-IV				
Fluid reasoning composite score	1 [26]	656	MD, F (NA)	1.02 (–2.43, 4.47)
Fluid reasoning composite score <85	1 [26]	656	RR, F (NA)	0.88 (0.66, 1.17)
Working memory composite score	1 [26]	656	MD, F (NA)	2.31 (–1.05, 5.67)
Working memory composite score <85	1 [26]	656	RR, F (NA)	0.89 (0.65, 1.22)
Processing speed primary index scale score^1^	1 [26]	656	MD, F (NA)	2.25 (–0.90, 5.40)
Processing speed primary index scale score^1^ <85	1 [26]	656	RR, F (NA)	0.89 (0.65, 1.22)
General ability primary index scale score	1 [26]	656	MD, F (NA)	3.36 (0.30, 6.42)^2^
General ability primary index scale score <85	1 [26]	656	RR, F (NA)	0.75 (0.56, 1.00)^2^
Cognitive proficiency primary index scale score	1 [26]	656	MD, F (NA)	2.55 (–1.05, 6.15)
Cognitive proficiency primary index scale score <85	1 [26]	656	RR, F (NA)	0.89 (0.67, 1.18)
WASI				
PIQ	1 [18]	196	MD, F (NA)	–0.55 (–4.28, 3.18)
PIQ < 85	1 [18]	196	RR, F (NA)	1.12 (0.58, 2.16)
Mild impairment: WPPSI-IV FSIQ or WASI FSIQ <85	2 [18,26]	852	RR, F (0%)	0.79 (0.62, 1.00)^2^
Moderate/severe impairment: WPPSI-IV FSIQ <70	1 [26]	656	RR, F (NA)	0.60 (0.33, 1.09)
ASQ: problem-solving score	1 [21]	132	MD, F (NA)	–2.70 (–5.90, 0.50)
Intellectual disability	1 [27]	715	RR, F (NA)	1.23 (0.60, 2.52)
Language				
ASQ: communication score	1 [21]	132	MD, IV, F (NA)	1.80 (–2.10, 5.70)
Motor				
ASQ				
Fine motor score	1 [21]	132	MD, F (NA)	–0.80 (–5.60, 4.00)
Gross motor score	1 [21]	132	MD, F (NA)	2.10 (–1.90, 6.10)
Cerebral palsy	1 [27]	715	RR, F (NA)	1.00 (0.54, 1.85)
Behavioral and emotional functioning				
SDQ				
Emotional symptoms score	3 [18,21,27]	1289	MD, R (0%)	0.16 (–0.11, 0.42)
Conduct problems score	3 [18,21,27]	1289	MD, R (0%)	–0.22 (–0.47, 0.02)
Hyperactivity/inattention score	3 [18,21,27]	1289	MD, R (0%)	–0.05 (–0.33, 0.22)
Peer relationship problems score	3 [18,21,27]	1289	MD, R (68%)	–0.09 (–0.49, 0.31)
Prosocial behavior score	3 [18,21,27]	1288	MD, R (0%)	–0.05 (–0.30, 0.20)
Impact score	2 [18,27]	1155	MD, F (0%)	0.08 (–0.18, 0.35)
Total difficulties score	3 [18,21,27]	1289	MD, R (0%)	–0.11 (–0.84, 0.62)
BRIEF				
Inhibit scale	3 [18,21,27]	1287	MD, R (0%)	0.22 (–1.33, 1.76)
Monitor scale	118	197	MD, F (NA)	1.68 (–2.16, 5.52)
Shift scale	3 [18,21,27]	1288	MD, R (47%)	0.78 (–1.33, 2.88)
Emotional control scale	3 [18,21,27]	1288	MD, R (0%)	0.33 (–1.41, 2.06)
Initiate scale	118	197	MD, F (NA)	0.51 (–3.10, 4.12)
Working memory scale	3 [18,21,27]	1287	MD, R (0%)	–0.69 (–2.53, 1.14)
Plan/organize scale	3 [18,21,27]	1285	MD, R (0%)	–0.27 (–2.00, 1.46)
Organization of materials scale	1 [18]	197	MD, F (NA)	–0.91 (–4.19, 2.37)
Inhibitory self-control index	1 [27]	958	MD, F (NA)	0.19 (–2.01, 2.39)
Flexibility index	2 [21,27]	1090	MD, F (0%)	0.18 (–1.59, 1.96)
Behavioral regulation index	2 [18,21]	329	MD, F (0%)	1.08 (–1.66, 3.81)
Metacognition index	2 [18,21]	329	MD, F (1%)	–1.03 (–3.73, 1.67)
Emergent metacognition index	1 [27]	958	MD, F (NA)	–0.01 (–2.49, 2.47)
Total score: global executive composite	3 [18,21,27]	1287	MD, R (0%)	–0.18 (–1.94, 1.58)
ASQ: personal-social score	1 [21]	132	MD, F (NA)	1.20 (–2.30, 4.70)
Autism spectrum disorder	1 [27]	715	RR, F (NA)	1.54 (0.90, 2.64)
Attention-deficit hyperactivity disorder or attention-deficit disorder	1 [27]	715	RR, F (NA)	1.10 (0.49, 2.47)
Other behavioral disorder	1 [27]	715	RR, F (NA)	1.01 (0.38, 2.66)

Test for heterogeneity represented by *I*^2^ statistic.

Abbreviations: ASQ, Ages and Stages Questionnaire; BRIEF, Behavior Rating Inventory of Executive Function; CI, confidence interval; F, fixed effects; FSIQ; full-scale intelligence quotient; IV, inverse variance; MD, mean difference; N, number of participants; NA, not applicable; PIQ, performance intelligence quotient; R, random effects; RCTs, randomized controlled trials; RR, risk ratio; SDQ, Strengths and Difficulties Questionnaire; WASI, Wechsler Abbreviated Scale of Intelligence; WPPSI, Wechsler Preschool and Primary Scale of Intelligence; VCC, verbal comprehension composite; VIQ, verbal intelligence quotient.

1 WPPSI-IV or WPPSI-III.

2 Statistically significant effect estimates.

*Language:* high-dose DHA was not associated with a difference in communication scores (ASQ) at CA of 5 y (1 RCT, 132 children).

*Motor:* there were no associations between high-dose DHA and motor scores (ASQ fine motor and gross motor) (1 RCT, 132 children) or cerebral palsy (1 RCT, 715 children) at CA of 5 y.

*Behavioral and emotional functioning:* high-dose DHA was not associated with differences in scores for emotional and behavioral functioning (SDQ) or executive functioning (BRIEF) at CA of 5–7 y (in ≤3 RCTs, 1289 children); nor for personal-social scores (ASQ) (1 RCT, 132 children), or diagnoses of autism spectrum disorder, attention-deficit hyperactivity disorder or attention disorder, or other behavioral disorders at CA of 5 y (all in 1 RCT, 715 children).

*Composite measure of impairment:* not reported by included RCTs.

#### Outcomes at 9–12 y

Not reported by included RCTs.

### Sensitivity analyses and publication bias

Prespecified sensitivity analyses were not conducted given the high quality of the RCTs. The number of included RCTs was not sufficient to assess publication bias through funnel plots.

## Discussion

In our systematic review and meta-analysis of 3 RCTs involving 2028 infants born at <29 wk of gestation, enteral high-dose DHA supplementation in the neonatal period was not associated with overall differences in global cognition scores at CA of 18–36 mo or 5–7 y. Although associations between high-dose DHA and most secondary outcomes were not seen, some potential benefits were observed. At CA of 18–36 m, high-dose DHA compared with control was associated with a reduction in mild motor impairment. Furthermore, at CA of 5–7 y, high-dose DHA was associated with a reduction in mild cognitive impairment, a higher general ability primary index scale score, and a reduction in scores <85 on this scale. Importantly, no negative impact on any neurodevelopmental outcome was observed.

Although our review’s findings are consistent with recent narrative reviews and DHA recommendations for preterm infants [[6], [7], [8]], they are limited by clinical heterogeneity in the characteristics of participants, interventions, and outcome assessments. Although we attempted to explore DHA regimen variation through formal subgroup analyses, our ability to do so was limited by the inclusion of only 3 RCTs reporting aggregate data. Although statistically no clear differential treatment effect based on supplement delivery method was discernible for our primary outcome, statistical heterogeneity was observed at CA of 5–7 y. The RCT (N3RO) that individually demonstrated benefit for this outcome (specifically, FSIQ at CA of 5 y [26]) used a direct enteral emulsion. This ensured that the full intended DHA dose (60 mg/kg/d) was achieved from intervention commencement [22]. The second RCT (DINO), which did not demonstrate benefit (CA of 7 y) [18], used enriched breast milk or preterm formula [16]. Authors of this RCT reported that although mean concentration of DHA in the breastmilk of mothers in the high-dose DHA group reached the intended 1% of total fats, the variation was wide [28], and the full available dose of DHA would not have been received until infants achieved full enteral feeds (∼ 18–20 d on average) [16]. DHA status of the infants in this RCT also varied at the end of the intervention, so that there was some overlap between the randomization groups [28], and this RCT was likely underpowered for this neurodevelopmental outcome (*N* = 196) [18]. The most recent RCT (MOBYDIck) used enriched breast milk and likely would have seen variation in DHA dose received by infants, although this was not assessed [19].

Though our review’s eligibility criteria enabled inclusion of RCTs assessing DHA in conjunction with other LCPUFAs, we did not identify any for inclusion at present. Infants in the RCTs were primarily provided mothers’ own milk, with some use of preterm infant formula or donor milk, all of which contain some arachidonic acid (AA). The potential importance of combining supplemental DHA with AA, and achieving DHA/AA “balance,” to maximize benefits and minimize harms of LCPUFAs, is being explored [8]. To date, 2 RCTs have evaluated the effects of combined DHA (50 mg/kg/d) and AA (100 mg/kg/d) supplementation in the neonatal period specifically for infants born at <29 wk of gestation. One observed a reduced risk of severe retinopathy of prematurity [29], but no effect on visual acuity at 2.5 y [30]; the other demonstrated improved brain maturation (specifically white matter microstructure) at term equivalent age [31]; however, follow-up to determine functional benefit is not yet available.

Review findings support current recommendations that preterm infants born at < 29 wk of gestation receive ∼50 mg/kg/d DHA, to approximate the estimated fetal accumulation rate [[6], [7], [8]]. Further research on the longer-term potential benefits and harms of high-dose DHA including follow-up of children into adolescence and adulthood is needed; along with research to situate neurodevelopmental benefits in the context of other short- and longer-term benefits or harms, such as the possible reduction and increase in severe retinopathy of prematurity [29] and bronchopulmonary dysplasia, respectively [32,33]. Future RCTs should explore variation in effects according to participant and treatment characteristics, use robust methodology, and aim for consistency in neurodevelopmental outcome measurement and reporting, to facilitate pooling of data.

This review has several limitations. The supplementation strategies differed between included RCTs, and the DHA dose received by infants in 2 RCTs was unclear, due to known variation in breastmilk DHA, even with DHA supplementation [28,34]. Although over 2000 infants born at <29 wk were randomly assigned in the included RCTs, the numbers of infants assessed for neurodevelopmental outcomes was far fewer. Thus, for review outcomes, infants analyzed ranged from 132 to 1289 in 1–3 RCTs, limiting statistical power. Additionally, although randomly assigned groups were well balanced in included RCTs, 2 RCTs identified baseline characteristic differences between parents of children who participated, and did not participate, in follow-up. For example, parents of children who completed follow-up, compared with those who did not, had higher levels of education [21,24], which may have contributed to an underestimation of effects. Furthermore, the inclusion of only 3 RCTs limited our ability to assess publication bias and statistically evaluate sources of heterogeneity. Behavioral and emotional functioning and composite measures of neurodevelopmental impairment were not reported by included RCTs at 1–3 and 4–8 y, respectively. No included RCT reported on neurodevelopment <1 y or at 9–12 y.

All RCTs were conducted across high-income countries and commenced recruitment between 2001 and 2015. Thus, applicability to low- and middle-income countries, and generalizability to present-day clinical context or practice should also be considered.

In conclusion, in our systematic review and meta-analysis of 3 RCTs, enteral high-dose DHA for infants born at <29 wk of gestation was not associated with overall differences in global cognition scores; however, benefit was observed in the largest RCT, which delivered DHA via a direct emulsion. Associations with reduced mild motor and cognitive impairment were also seen, and no negative impacts were observed. Together, neurodevelopmental outcome results support current recommendations regarding high-dose DHA for extremely preterm infants. Further research is required to understand variation by participant and treatment characteristics, and to situate these associations in the context of other short- and longer-term outcomes.

## Author contributions

The authors’ responsibilities were as follows – ES, NI, MM, JG: designed research; ES, NI, JG: conducted research; ES: analyzed data, drafted manuscript, and had primary responsibility for final content; and all authors: interpreted data, critically reviewed manuscript, and reviewed and approved the final manuscript.

## Data availability

The authors confirm that the data supporting the findings of this study are available within the article (and/or) its supplementary materials.

## Funding

Emily Shepherd (ID 2007800), Thomas Sullivan (ID APP1173576), Maria Makrides (ID GNT 2016756), and Jacqueline Gould (ID GNT 2033062) are in receipt of Australian National Health and Medical Research Council Investigator Grants (https://www.nhmrc.gov.au/). The funder had no role in study design, data collection and analysis, decision to publish, or preparation of the manuscript.

## Conflict of interest

TRS, IM, MG, AM, RG, MM, and JG report being authors on publication(s) included in this review. ES and NI have no conflicts of interest relevant to this article to disclose.
