# Supplementary material for: Enteral High-Dose Docosahexaenoic Acid and Neurodevelopment in Extremely Preterm Infants: A Systematic Review and Meta-analysis
Source: Curr Dev Nutr. 2025 Jul 24;9(9):107510. doi: 10.1016/j.cdnut.2025.107510 (PMC12396270; doi:10.1016/j.cdnut.2025.107510)
Supplement: Multimedia component 1 [file mmc1.docx]

**Supplemental Material**

**Supplemental Tables**

**Supplementary Table 1. *CINAHL* Search Strategy and Results**

| **#** | **Search strategy** (9 December 2022) | **Results** |
| --- | --- | --- |
| 1 | (“Randomized Controlled Trials” OR MH “Double-Blind Studies” OR MH “Single-Blind Studies” OR MH “Random Assignment” OR MH “Pretest-Posttest Design” OR MH “Cluster Sample” OR TI (Randomised OR Randomized) OR AB (Random*) OR TI (Trial) OR (MH (“Sample Size”) AND AB (Assigned OR Allocated OR Control)) OR MH (Placebos) OR PT (“Randomized Controlled Trial”) OR AB (Control W5 Group) OR MH (“Crossover Design”) OR MH (“Comparative Studies”) OR AB (Cluster W3 RCT)) NOT ((MH “Animals+” OR MH “Animal Studies” OR TI “Animal Model*”) NOT MH “Human”) | 941,068 |
| 2 | (MH “Fatty Acids, Omega-3” OR MH “Docosahexaenoic Acids+” OR MH “Fish Oils” OR MH “Fatty Acids, Essential) | 14,387 |
| 3 | (“omega 3” OR “n-3” OR “polyunsaturated fatty acid*” OR PUFA* OR LCPUFA* OR “docosahex#enoic acid” OR docosahex#enoate OR DHA OR “alga* oil*” OR “marine oil*” OR “fish oil*”) | 16,062 |
| 4 | S2 or S3 | 20,090 |
| 5 | (MH “Infant, Newborn” OR MH “Infant, Low Birth Weight+” OR MH “Infant, Premature” OR MH “Childbirth, Premature”) | 162,015 |
| 6 | (baby OR babies or neonat* OR infan* OR newborn* OR “new born” OR “preterm birth*” OR “premature birth*”) | 545,810 |
| 7 | S5 OR S6 | 546,790 |
| 8 | (MH “Child Development” OR MH “Child Development Disorders” OR MH “Neurologic Manifestations” OR MH “Psychomotor Performance” OR MH “Neurologic Examination” OR MH “Intelligence+” OR MH “Cognition+”) | 8,085 |
| 9 | (neuro* OR cogniti* OR psycho* OR intelligen* OR IQ OR ((language OR motor OR movement OR behaviour$) N2 (develop* OR impair* OR disorder*))) | 1,480,599 |
| 10 | S8 OR S9 | 1,481,153 |
| 11 | S1 AND S4 AND S7 AND S10 | 227 |
| *Top up of search strategy (15 January 2024)* | | 9 |
| *Top up of search strategy (4 November 2024)* | | 5 |
| **Total** | | 241 |

**Supplementary Table 2. *Cochrane Library* Search Strategy and Results**

| **#** | **Search strategy** (9 December 2022) | **Results** |
| --- | --- | --- |
| 1 | MeSH descriptor: [Fatty Acids, Omega-3] this term only | 2,140 |
| 2 | MeSH descriptor: [Docosahexaenoic Acids] explode all trees | 1,311 |
| 3 | MeSH descriptor: [Fish Oils] this term only | 1,107 |
| 4 | MeSH descriptor: [Fatty Acids, Essential] this term only | 171 |
| 5 | #1 OR #2 OR #3 OR #4 | 3,683 |
| 6 | (“omega 3” OR “n-3” OR “polyunsaturated NEXT fatty NEXT acid*” OR PUFA* OR LCPUFA* OR “docosahex?enoic NEXT acid” OR docosahex?enoate OR DHA OR “alga* NEXT oil*” OR “marine NEXT oil*” OR “fish NEXT oil*”):ti,ab | 11,109 |
| 7 | #5 OR #6 | 11,478 |
| 8 | MeSH descriptor: [Infant, Newborn] this term only | 17,631 |
| 9 | MeSH descriptor: [Infant, Low Birth Weight] explode all trees | 2,342 |
| 10 | MeSH descriptor: [Infant, Premature] explode all trees | 4,293 |
| 11 | MeSH descriptor: [Premature Birth] this term only | 1,826 |
| 12 | #8 OR #9 OR #10 OR #11 | 19,160 |
| 13 | (baby OR babies OR neonat* OR infan* OR newborn* OR “new born” OR (preterm NEXT birth*) OR (premature NEXT birth*)):ti,ab | 67,398 |
| 14 | #12 OR #13 | 71,108 |
| 15 | MeSH descriptor: [Child Development] explode all trees | 8,828 |
| 16 | MeSH descriptor: [Neurodevelopmental Disorders] explode all trees | 13,129 |
| 17 | MeSH descriptor: [Neurocognitive Disorders] explode all trees | 84,081 |
| 18 | MeSH descriptor: [Neurologic Manifestations] explode all trees | 9,448 |
| 19 | MeSH descriptor: [Psychomotor Performance] explode all trees | 24,986 |
| 20 | MeSH descriptor: [Neurologic Examination] explode all trees | 11,912 |
| 21 | MeSH descriptor: [Cognition] explode all trees | 2,786 |
| 22 | MeSH descriptor: [Intelligence] explode all trees | 6,563 |
| 23 | #15 OR #16 OR #17 OR #18 OR #19 OR #20 OR #21 OR #22 | 130,590 |
| 24 | (neuro* OR cogniti* OR psycho* OR intelligen* OR IQ):ti,ab OR ((language OR motor OR movement OR behaviour*) NEAR/2 (develop* OR impair* OR disorder*)):ti,ab | 247,676 |
| 25 | #23 OR #24 | 338,218 |
| 26 | #7 AND #14 AND #25 | 366 |
| *Top up of search strategy (15 January 2024)* | | 24 |
| *Top up of search strategy (4 November 2024)* | | 11 |
| **Total** | | 401 |

**Supplementary Table 3. *Embase and Medline* Search Strategy and Results**

| **#** | **Search strategy** (9 December 2022) | **Results** |
| --- | --- | --- |
| 1 | (((randomized controlled trial) or (controlled clinical trial)).pt. or (randomized or randomised or placebo or randomly or trial or groups).ab.) not (exp animals/ not humans.sh.) | 3,563,780 |
| 2 | Fatty Acids, Omega-3/ or exp Docosahexaenoic Acids/ or Fish Oils/ or Fatty Acids, Essential/ | 93,968 |
| 3 | ((omega 3) or (n-3) or (polyunsaturated fatty acid$) or PUFA$ or LCPUFA$ or (docosahex?enoic acid) or docosahex?enoate or DHA or (alga$ oil$) or (marine oil$) or (fish oil$)).ti,ab. | 300,132 |
| 4 | 2 or 3 | 325,303 |
| 5 | Infant, Newborn/ or exp Infant, Low Birth Weight/ or exp Infant, Premature or Premature Birth/ | 1,303,857 |
| 6 | (baby or babies or neonat$ or infan$ or newborn$ or (new born) or (preterm birth$) or (premature birth$)).ti,ab. | 1,849,572 |
| 7 | 5 or 6 | 2,390,795 |
| 8 | exp Child Development/ or exp Neurodevelopmental Disorders/ or exp Neurocognitive Disorders/ or exp Neurologic manifestations/ or exp Psychomotor Performance/ or Neurologic Examination/ or exp Intelligence/ or exp Cognition/ | 9,700,827 |
| 9 | (neuro$ or cogniti$ or psycho$ or intelligen$ or IQ).ti,ab. or ((language or motor or movement or behaviour$) adj2 (develop$ or impair$ or disorder$)).ti,ab. | 7,038,788 |
| 10 | 8 or 9 | 13,417,321 |
| 11 | 1 and 4 and 7 and 10 | 506 |
| 12 | remove duplicates from 11 | 497 |
| *Top up of search strategy (15 January 2024)* | | 63 |
| *Top up of search strategy (4 November 2024)* | | 21 |
| **Total** | | 545 |

**Supplementary Table 4. *Scopus* Search Strategy and Results**

| **#** | **Search strategy** | **Results** |
| --- | --- | --- |
| 1 | ABS (randomized OR randomised OR placebo OR randomly OR trial OR groups)  AND  ABS (baby OR babies OR neonat* OR infan* OR newborn* OR “new born” OR “preterm birth*” OR “premature birth*”)  AND  ABS (“omega 3” OR “polyunsaturated fatty acid*” OR PUFA* OR LCPUFA* OR “docosahex?enoic acid” OR docosahex?enoate OR DHA OR “alga* oil*” OR “marine oil*” OR “fish oil*”)  AND  ABS (neuro* or cogniti* or psycho* or intelligen* or IQ) OR ((language OR motor OR movement OR behaviour$) NEAR/2 (develop* OR impair* OR disorder*)) | 268 |
| *Top up of search strategy (15 January 2024)* | | 17 |
| *Top up of search strategy (4 November 2024)* | | 12 |
| **Total** | | 297 |

**Supplementary Table 5. *Web of Science* Search Strategy and Results**

| **#** | **Search strategy** | **Results** |
| --- | --- | --- |
| 1 | AB=(randomized OR randomised OR placebo OR randomly OR trial OR groups) | 6,710,980 |
| 2 | AB=(baby OR babies OR neonat* OR infan* OR newborn* OR “new born” OR “preterm birth*” OR “premature birth*”) | 572,512 |
| 3 | AB=(“omega 3” OR “n-3” OR “polyunsaturated fatty acid*” OR PUFA* OR LCPUFA* OR “docosahex?enoic acid” OR docosahex?enoate OR DHA OR “alga* oil*” OR “marine oil*” OR “fish oil*”) | 213,710 |
| 4 | AB=(neuro* or cogniti* or psycho* or intelligen* or IQ) OR ((language OR motor OR movement OR behaviour$) NEAR/2 (develop* OR impair* OR disorder*)) | 3,254,657 |
| 5 | #1 AND #2 AND #3 AND #4 | 535 |
| 6 | #1 AND #2 AND #3 AND #4 and Review Article or Editorial Material or Book Chapters (Exclude – Document Types) | 449 |
| *Top up of search strategy (15 January 2024)* | | 24 |
| *Top up of search strategy (4 November 2024)* | | 21 |
| **Total** | | 494 |

**Supplementary Table 6. Reports Excluded at Full Text Review**

| **No.** | **Citation** | **Reason for exclusion** |
| --- | --- | --- |
| 1 | Almaas AN, Tamnes CK, Nakstad B, et al. Diffusion tensor imaging and behavior in premature infants at 8 years of age, a randomized controlled trial with long-chain polyunsaturated fatty acids. *Early Hum Dev*. 2016;95:41-46. | Wrong participants* |
| 2 | Almaas AN, Tamnes CK, Nakstad B, et al. Long-chain polyunsaturated fatty acids and cognition in VLBW infants at 8 years: an RCT. *Pediatrics*. 2015;135(6):972-980. | Wrong participants* |
| 3 | Andrew M, Parr J, Montague-Johnson C, et al. Optimising nutrition to improve growth and reduce neurodisabilities in neonates at risk of neurological impairment. *Dev Med Child Neurol.* 2016;58(S5):23‐24. | Wrong participants* |
| 4 | Andrew M, Parr JR, Montague-Johnson C, et al. Optimising nutrition to improve growth and reduce neurodisabilities in neonates at risk of neurological impairment. *J Pediatr Gastroenterol Nutr*. 2016;62(Supp 1):665. | Wrong participants* |
| 5 | Andrew MJ, Montague‐Johnson C, Laler K, et al. Neurodevelopmental outcome of nutritional intervention in newborn infants at risk of neurodevelopmental impairment: the Dolphin neonatal double-blind randomized controlled trial. *Dev Med Child Neurol*. 2018;60(9):897-905. | Wrong participants* |
| 6 | Andrew MJ, Parr JR, Montague-Johnson C, Baker BL, Holmes J, Laler K, et al. Optimising nutrition to improve growth and reduce neurodisabilities in neonates at risk of neurological impairment. *Arch Dis Child*. 2016;101(Suppl 1):A21. | Wrong participants* |
| 7 | Ashorn P, Alho L, Ashorn U, et al. Supplementation of maternal diets during pregnancy and for 6 months postpartum and infant diets thereafter with small-quantity lipid-based nutrient supplements does not promote child growth by 18 months of age in rural Malawi: a randomized controlled trial. *J Nutr.* 2015;145(6):1345-1353. | Wrong participants |
| 8 | Baack ML, Puumala SE, Messier SE, Pritchett DK, Harris WS. Daily enteral DHA supplementation alleviates deficiency in premature infants. *Lipids*. 2016;51(4):423-433. | Wrong outcomes |
| 9 | Baack ML. Bridging the docosahexaenoic acid (DHA) gap: the effects of omega-3 fatty acid supplementation in premature infants. ClinicalTrials.gov identifier: NCT01908907. Updated March 21, 2019. Accessed July 2, 2024. <https://clinicaltrials.gov/show/NCT01908907> | Trial registration |
| 10 | Beyerlein A, Hadders-Algra M, Kennedy K, et al. Infant formula supplementation with long-chain polyunsaturated fatty acids has no effect on Bayley developmental scores at 18 months of age--IPD meta-analysis of 4 large clinical trials. *J Pediatr Gastroenterol Nutr*. 2010;50(1):79-84. | Wrong study design |
| 11 | Bouglé D, Denise P, Vimard F, Nouvelot A, Penneillo MJ, Guillois B. Early neurological and neuropsychological development of the preterm infant and polyunsaturated fatty acids supply. *Clin Neurophysiol.* 1999;110(8):1363‐1370. | Wrong participants* |
| 12 | Carlson SE. Kansas University DHA outcome study (KUDOS) follow-up. ClinicalTrials.gov identifier: NCT02487771. Updated February 7, 2018. Accessed July 2, 2024. <https://clinicaltrials.gov/show/NCT02487771> | Trial registration |
| 13 | Carnielli VP, Simonato M, Verlato G, et al. Synthesis of long-chain polyunsaturated fatty acids in preterm newborns fed formula with long-chain polyunsaturated fatty acids. *Am J Clin Nutr*. 2007;86(5):1323‐1330. | Wrong participants* |
| 14 | Corvaglia L. Krill oil supplementation: effects on breast milk composition. ClinicalTrials.gov identifier: NCT03583502. Updated July 11, 2018. Accessed July 2, 2024. <https://clinicaltrials.gov/show/NCT03583502> | Trial registration |
| 15 | Castillo FS. Enteral supplementation with docosahexaenoic acid and arachidonic acid (DHA-AA) in preterm infants. ClinicalTrials.gov identifier: NCT06366893. Updated April 19, 2024. Accessed November 6, 2024. https://clinicaltrials.gov/study/NCT06366893 | Trial registration |
| 16 | Cheatham CL, Nerhammer AS, Asserhøj M, Michaelsen KF, Lauritzen L. Fish oil supplementation during lactation: Effects on cognition and behavior at 7 years of age. *Lipids*. 2011;46(7):637-645. | Wrong participants |
| 17 | Cimatti AG, Martini S, Munarini A, et al. Maternal supplementation with Krill Oil during breastfeeding and long-chain polyunsaturated fatty acids (LCPUFAs) composition of human milk: A feasibility study. *Front Pediatr.* 2018;6:407. | Wrong participants |
| 18 | Clandinin MT, Van Aerde JE, Merkel KL, et al. Growth and development of preterm infants fed infant formulas containing docosahexaenoic acid and arachidonic acid. *J Pediatr.* 2005;146(4):461-468. | Wrong participants* |
| 19 | Collins CT, Gibson RA, Makrides M, et al. The N3RO trial: a randomised controlled trial of docosahexaenoic acid to reduce bronchopulmonary dysplasia in preterm infants < 29 weeks' gestation. *BMC Pediatr.* 2016;16:72. | Additional paper for included trial |
| 20 | Collins CT, Gibson RA, McPhee AJ, Makrides M. Neurodevelopmental outcomes at 7-years' corrected age in preterm infants fed high-dose docosahexaenoic acid to term equivalent: a follow-up of the DINO randomised controlled trial. *J Paediatr Child Health*. 2015;51(S1):57. | Additional abstract for included trial |
| 21 | Collins CT, Sullivan TR, McPhee AJ, Stark MJ, Makrides M, Gibson RA. A dose response randomised controlled trial of docosahexaenoic acid (DHA) in preterm infants. *Prostaglandins Leukot Essent Fatty Acids*. 2015;99:1‐6. | Wrong outcomes |
| 22 | Colombo J, Carlson SE, Cheatham CL, et al. Long-term effects of LCPUFA supplementation on childhood cognitive outcomes. *Am J Clin Nutr.* 2013;98(2):403-412. | Wrong participants |
| 23 | Colombo J, Jill Shaddy D, Kerling EH, Gustafson KM, Carlson SE. Docosahexaenoic acid (DHA) and arachidonic acid (ARA) balance in developmental outcomes. *Prostaglandins Leukot Essent Fatty Acids*. 2017;121:52-56. | Wrong participants |
| 24 | Colombo J. LCPUFA supplementation in infancy affects measures of preschool cognition. Pediatric Academic Societies 2011 Annual Meeting, Denver, Colorado, USA. 2011. | Wrong participants |
| 25 | Delgado-Noguera MF, Calvache JA, Bonfill Cosp X, Kotanidou EP, Galli-Tsinopoulou A. Supplementation with long chain polyunsaturated fatty acids (LCPUFA) to breastfeeding mothers for improving child growth and development. *Cochrane Database Syst Rev.* 2015(7):CD007901. | Wrong study design |
| 26 | Drevon CA. A randomized clinical trial on supplementation of DHA and AA to preterm infants. ClinicalTrials.gov identifier: NCT00226187. Updated February 15, 2007. Accessed July 2, 2024.  <https://clinicaltrials.gov/show/NCT00226187> | Trial registration |
| 27 | Drover JR, Felius J, Hoffman DR, et al. A randomized trial of DHA intake during infancy: School readiness and receptive vocabulary at 2-3.5 years of age. Early Hum Dev. 2012;88(11):885-891. | Wrong participants |
| 28 | Eilander A, Hundscheid DC, Osendarp SJ, Transler C, Zock PL. Effects of n-3 long chain polyunsaturated fatty acid supplementation on visual and cognitive development throughout childhood: A review of human studies. *Prostaglandins Leukotr Essent Fatty Acids*. 2007;76(4):189-203. | Wrong study design |
| 29 | Farshbaf-Khalili A. The effect of fish-oil supplementation on pregnancy outcomes in mother and infant. WHO International Clinical Trials Registry identifier: IRCT2013100914957N1. Updated February 22, 2018. Accessed July 2, 2024. <https://trialsearch.who.int/Trial2.aspx?TrialID=IRCT2013100914957N1> | Trial registration |
| 30 | Fewtrell MS, Morley R, Abbott RA, et al. Double-blind, randomized trial of long-chain polyunsaturated fatty acid supplementation in formula fed to preterm infants. *Pediatrics*. 2002;110(1 Pt 1):73-82. | Wrong participants* |
| 31 | Firouzabadi FD, Shab-Bidar S, Jayedi A. The effects of omega-3 polyunsaturated fatty acids supplementation in pregnancy, lactation, and infancy: An umbrella review of meta-analyses of randomized trials. Pharmacol Res. 2022;177:106100. | Wrong study design |
| 32 | Foreman-van Drongelen MM, van Houwelingen AC, Kester AD, Blanco CE, Hasaar TH, Hornstra G. Influence of feeding artificial-formula milks containing docosahexaenoic and arachidonic acids on the postnatal long-chain polyunsaturated fatty acid status of healthy preterm infants. *Br J Nutr.* 1996;76(5):649‐667. | Wrong participants* |
| 33 | Ghraf R, Jelinek J, Lehwalder D. Status of clinical trials with the long-chain polyunsaturated fatty acid infant formula Aptamil with Milupan. *Eur J Clin Nutr.* 1994;48(Suppl 2):S31‐34. | Wrong participants* |
| 34 | Gould JF, Makrides M, Sullivan TR, et al. IQ at 5-years' corrected age: a follow-up of the N3RO randomised controlled trial of docosahexaenoic acid in preterm infants born <29 weeks' gestation. *J Paediatr Child Health*. 2022;58(S2):162. | Additional abstract for included trial |
| 35 | Gould J, Makrides M, Sullivan T, Bednarz J, Gibson R, McPhee A. A randomised controlled trial of docosahexaenoic acid in preterm infants born before 29 weeks’ gestation and IQ at 5 years. J Pediatr Gastroenterol Nutr 2023; 76(S1): 982. | Additional abstract for included trial |
| 36 | Guillot M, Robitaille CA, Turner L, et al. Effects of maternal docosahexaenoic acid supplementation on brain development and neurodevelopmental outcomes of breastfed preterm neonates: protocol for a follow-up at preschool age of a randomised clinical trial (MOBYDIckPS). *BMJ Open*. 2022;12(5):e057482. | Additional protocol for included trial |
| 37 | Harris M, Stacy M, Baker S, McGirr K, Davalos D. The omega smart baby project: effect of maternal DHA on infant development. *FASEB J*. 2014;28(S1)269.1. | Wrong participants |
| 38 | Helland IB, Saugstad OD, Smith L, et al. Similar effects on infants of n-3 and n-6 fatty acids supplementation to pregnant and lactating women. *Pediatrics*. 2001;108(5):E82. | Wrong participants |
| 39 | Helland IB, Smith L, Blomen B, Saarem K, Saugstad OD, Drevon CA. Effect of supplementing pregnant and lactating mothers with n-3 very-long-chain fatty acids on children's IQ and body mass index at 7 years of age. *Pediatrics*. 2008;122(2):e472-e479. | Wrong participants |
| 40 | Helland IB, Smith L, Saarem K, Saugstad OD, Drevon CA. Maternal supplementation with very-long-chain n-3 fatty acids during pregnancy and lactation augments children's IQ at 4 years of age. *Pediatrics*. 2003;111(1):e39-e44. | Wrong participants |
| 41 | Henriksen C, Almaas A, Westerberg A, et al. Growth, metabolic markers, and cognition in 8-year old children born prematurely, follow-up of a randomized controlled trial with essential fatty acids. *Eur J Pediatr.* 2016;175(9):1165-1174. | Wrong participants* |
| 42 | Henriksen C, Haugholt K, Lindgren M, et al. Improved cognitive development among preterm infants attributable to early supplementation of human milk with docosahexaenoic acid and arachidonic acid. *Pediatrics*. 2008;121(6):1137‐1145. | Wrong participants* |
| 43 | Hu R, Zu J, Hua Y, Li Y, Li J. Could early life DHA supplementation benefit neurodevelopment? A systematic review and meta-analysis. *Front Neurol*. 2024;15:1295788. | Wrong study design |
| 44 | Hurtado JA, Iznaola C, Peña M, et al. Effects of maternal ω-3 supplementation on fatty acids and on visual and cognitive development. *J Pediatr Gastroenterol Nutr.* 2015;61(4):472‐480. | Wrong participants |
| 45 | Iversen PO. Nutrition, growth and development among very preterm infants. ClinicalTrials.gov identifier: NCT01103219. Updated May 3, 2017. Accessed July 2, 2024. <https://clinicaltrials.gov/show/NCT01103219> | Trial registration |
| 46 | Janson E, Koolschijn PCMP, Schipper L, et al. Dolphin CONTINUE: a multi-center randomized controlled trial to assess the effect of a nutritional intervention on brain development and long-term outcome in infants born before 30 weeks of gestation. *BMC Pediatr* 2024; 24:384. | Trial protocol |
| 47 | Jensen CL, Voigt RG, Llorente AM, et al. Effect of maternal docosahexaenoic acid (DHA) supplementation on neuropsychological and visual status of former breast-fed infants at five years of age. *J Pediatr Gastroenterol Nutr*. 2004;39(Suppl 1):S10. | Wrong participants |
| 48 | Jensen CL, Voigt RG, Prager TC, et al. Effects of maternal docosahexaenoic acid (DHA) supplementation on visual function and neurodevelopment of breast-fed infants. *Pediatr Res.* 2001;49(4):448A. | Wrong participants |
| 49 | Kaempf-Rotzoll DE, Hellstern G, Linderkamp O. Influence of long-chain polyunsaturated fatty acid formula feeds on vitamin E status in preterm infants. *Int J Vitam Nutr Res*. 2003;73(5):377-387. | Wrong participants* |
| 50 | Keim S. Omega tots long term follow-up. ClinicalTrials.gov identifier: NCT05191823. Updated December 20, 2023. Accessed July 2, 2024. <https://clinicaltrials.gov/show/NCT05191823> | Trial registration |
| 51 | Khandelwal S, Kondal D, Chaudhry M, et al. Effect of maternal docosahexaenoic acid (DHA) supplementation on offspring neurodevelopment at 12 months in India: a randomized controlled trial. *Nutrients*. 2020;12(10):3041. | Wrong participants |
| 52 | Khandelwal S, Swamy MK, Patil K, et al. The impact of DocosaHexaenoic Acid supplementation during pregnancy and lactation on Neurodevelopment of the offspring in India (DHANI): trial protocol. *BMC Pediatr*. 2018;18(1):261. | Trial protocol |
| 53 | Lauritzen L, Eriksen SE, Hjorth MF, et al. Maternal fish oil supplementation during lactation is associated with reduced height at 13 years of age and higher blood pressure in boys only. *Br J Nutr*. 2016;116(12):2082-2090. | Wrong participants |
| 54 | Lauritzen L. Fish oil supplementation in lactation. ClinicalTrials.gov identifier: NCT00266305. Updated August 14, 2008. Accessed July 2, 2024. <https://clinicaltrials.gov/study/NCT00266305> | Trial registration |
| 55 | Lehner A, Staub K, Aldakak L, et al. Impact of omega-3 fatty acid DHA and EPA supplementation in pregnant or breast-feeding women on cognitive performance of children: systematic review and meta-analysis. *Nutr Rev*. 2021;79(5):585-598. | Wrong study design |
| 56 | Lepping RJ, Honea RA, Martin LE, et al. Long-chain polyunsaturated fatty acid supplementation in the first year of life affects brain function, structure, and metabolism at age nine years. *Dev Psychobiol*. 2019;61(1):5-16. | Wrong participants |
| 57 | Liao K, McCandliss BD, Carlson SE, et al. Event-related potential differences in children supplemented with long-chain polyunsaturated fatty acids during infancy. *Dev Sci*. 2017;20(5):e12455. | Wrong participants |
| 58 | Lucas A, Stafford M, Morley R, et al. Efficacy and safety of long-chain polyunsaturated fatty acid supplementation of infant-formula milk: a randomised trial. *Lancet.* 1999;354(9194):1948-1954. | Wrong participants |
| 59 | Makrides, Gibson, Collins, McPhee. The DINO trial outcomes: does high-dose dietary docosahexaenoic acid (DHA) improve the neurodevelopmental outcome of preterm infants? International Society for the Study of Fatty Acids and Lipids (ISSFAL) 8th International Congress, Kansas City, Missouri, USA. 2008: Abstract no: P11. | Additional abstract for included trial |
| 60 | Moon K, Rao SC, Schulzke SM, Patole SK, Simmer K. Longchain polyunsaturated fatty acid supplementation in preterm infants. *Cochrane Database Syst Rev.* 2016;12:CD000375. | Wrong study design |
| 61 | Modi N. Exploratory randomised double-blind controlled trial of breast milk fortifier with and without long chain polyunsaturated fatty acid (LCPUFA) supplementation on body composition in preterm infants. WHO International Clinical Trials Registry identifier: ISRCTN59878178. Updated January 13, 2015. Accessed July 2, 2024. <https://trialsearch.who.int/Trial2.aspx?TrialID=ISRCTN59878178> | Trial registration |
| 62 | Nevins JEH, Donovan SM, Snetselaar L, et al. Omega-3 fatty acid dietary supplements consumed during pregnancy and lactation and child neurodevelopment: a systematic review. *J Nutr*. 2021;151(11):3483-3494. | Wrong study design |
| 63 | Nieto-Ruiz A, Dieguez E, Sepulveda-Valbuena N, et al. The effects of an infant formula enriched with milk fat globule membrane, long-chain polyunsaturated fatty acids and synbiotics on child behavior up to 2.5 years old: the COGNIS study. *Nutrients*. 2020;12(12):3825. | Wrong participants |
| 64 | O'Connor DL, Hall R, Adamkin Det al. Growth and development in preterm infants fed long-chain polyunsaturated fatty acids: a prospective, randomized controlled trial. *Pediatrics*. 2001;108(2):359‐371. | Wrong participants* |
| 65 | O'Connor DL, Jacobs F, Hall R, et al. Growth and development of premature infants fed predominantly human milk, predominantly premature infant formula, or a combination of human milk and premature formula. *J of Pediatr Gastroenterol Nutr.* 2003;37(4):437-446. | Wrong participants* |
| 66 | Ochoa JJ. Effect of mother DHA supplementation on premature newborn. ClinicalTrials.gov identifier: NCT01940640. Updated December 2, 2015. Accessed July 2, 2024. <https://clinicaltrials.gov/show/NCT01940640> | Trial registration |
| 67 | Ostadrahimi A, Salehi-Pourmehr H, Mohammad-Alizadeh-Charandabi S, Heidarabady S, Farshbaf-Khalili A. The effect of perinatal fish oil supplementation on neurodevelopment and growth of infants: a randomized controlled trial. *Eur J Nutr.* 2018;57(7):2387-2397. | Wrong participants |
| 68 | Schulzke SM, Patole SK, Simmer K. Long-chain polyunsaturated fatty acid supplementation in preterm infants. *Cochrane Database Syst Rev*. 2011(2):CD000375. | Wrong study design |
| 69 | See VHL, Mori TA, Prescott SL, Beilin LJ, Burrows S, Huang RC. Cardiometabolic risk factors at 5 years after omega-3 fatty acid supplementation in infancy. *Pediatrics*. 2018;142(1):e20162623. | Wrong participants |
| 70 | Shulkin M, Pimpin L, Bellinger D, et al. N-3 fatty acid supplementation in mothers, preterm infants, and term infants and childhood psychomotor and visual development: a systematic review and meta-analysis. *J Nutr.* 2018;148(3):409-418. | Wrong study design |
| 71 | Simmer K. Long-chain polyunsaturated fatty acid supplementation in preterm infants. *Cochrane Database Syst Rev.* 2000(2):CD000375. | Wrong study design |
| 72 | Smithers LG, Collins CT, Simmonds LA, Gibson RA, McPhee A, Makrides M. Feeding preterm infants milk with a higher dose of docosahexaenoic acid than that used in current practice does not influence language or behavior in early childhood: a follow-up study of a randomized controlled trial. *Am J Clin Nutr.* 2010;91(3):628-634. | Additional paper for included trial |
| 73 | Smithers LG, Collins CT, Simmonds LA, Gibson RA, McPhee AJ, Makrides M. Higher-dose docosahexaenoic acid (DHA) does not influence language or behaviour: A follow up of the DINO (DHA for the improvement of neurodevelopmental outcome in preterm infants) trial. *J Paediatr Child Health.* 2010;46(S1):39. | Additional abstract for included trial |
| 74 | Smithers LG, Gibson RA, McPhee A, Makrides M. Effect of long-chain polyunsaturated fatty acid supplementation of preterm infants on disease risk and neurodevelopment: a systematic review of randomized controlled trials. *Am J Clin Nutr.* 2008;87(4):912-920. | Wrong study design |
| 75 | Stalker V. Developmental outcomes of long-term feed supplementation in newborn babies. WHO International Clinical Trials Registry identifier: ISRCTN62323236. Updated November 28, 2023. July 2, 2024. <https://trialsearch.who.int/Trial2.aspx?TrialID=ISRCTN62323236> | Trial registration |
| 76 | Stiris T. Nutrition therapy in the immature infant (ImNuT). ClinicalTrials.gov identifier: NCT03555019. Updated September 8, 2021. Accessed July 2, 2024. <https://clinicaltrials.gov/show/NCT03555019> | Trial registration |
| 77 | Sullivan P. Optimising nutrition to improve growth and reduce neurodisabilities in neonates at risk of neurological impairment. WHO International Clinical Trials Registry identifier: ISRCTN39264076. Updated February 11, 2019. July 2, 2024. <https://trialsearch.who.int/Trial2.aspx?TrialID=ISRCTN39264076> | Trial registration |
| 78 | Unknown [University of Oslo]. Nutrition, growth and development among very preterm infants – PRENU. WHO International Clinical Trials Registry identifier: EUCTR2010-020464-38-NO. Updated March 19, 2012. Accessed July 2, 2024. <https://trialsearch.who.int/Trial2.aspx?TrialID=EUCTR2010-020464-38-NO> | Trial registration |
| 79 | Unknown [University of Oslo]. The effects of the fatty acids arachidonic acid (ARA) and docosahexaenoic acid (DHA) on growth, metabolism and inflammatory response in infants born extremely preterm. WHO International Clinical Trials Registry identifier: EUCTR2016-003700-31-NO. Updated December 11, 2017. Accessed July 2, 2024. <https://trialsearch.who.int/Trial2.aspx?TrialID=EUCTR2016-003700-31-NO> | Trial registration |
| 80 | Unknown [Ain Shams University]. Effect of DHA on proinflammatory cytokines including platelets activating factor (PAT) in preterm neonates. ClinicalTrials.gov identifier: NCT04746885. Updated February 10, 2021. Accessed July 2, 2024. <https://clinicaltrials.gov/show/NCT04746885> | Trial registration |
| 81 | van Goor SA, Dijck-Brouwer DA, Erwich JJ, Schaafsma A, Hadders-Algra M. The influence of supplemental docosahexaenoic and arachidonic acids during pregnancy and lactation on neurodevelopment at eighteen months. *Prostaglandins Leukotr Essent Fatty Acids*. 2011;84(5-6):139-146. | Wrong participants |
| 82 | van Wezel-Meijler G, van der Knaap MS, Huisman J, Jonkman EJ, Valk J, Lafeber HN. Dietary supplementation of long-chain polyunsaturated fatty acids in preterm infants: effects on cerebral maturation. *Acta Paediatr*. 2002;91(9):942-950. | Wrong participants* |
| 83 | Verfuerden ML, Dib S, Jerrim J, Fewtrell M, Gilbert RE. Effect of long-chain polyunsaturated fatty acids in infant formula on long-term cognitive function in childhood: a systematic review and meta-analysis of randomised controlled trials. *PLoS One.* 2020;15(11):e0241800. | Wrong study design |
| 84 | Verfurden ML, Gilbert R, Lucas A, Jerrim J, Fewtrell M. Effect of nutritionally modified infant formula on academic performance: linkage of seven dormant randomised controlled trials to national education data. *BMJ*. 2021;375:e065805. | Wrong study design |
| 85 | Wang Q, Cui Q, Yan C. The effect of supplementation of long-chain polyunsaturated fatty acids during lactation on neurodevelopmental outcomes of preterm infant from infancy to school age: a systematic review and meta-analysis. *Pediatr Neurol*. 2016;59:54-61.e51. | Wrong study design |
| 86 | Wendel K, Pfeiffer HCV, Fugelseth DM, et al. Effects of nutrition therapy on growth, inflammation and metabolism in immature infants: a study protocol of a double-blind randomized controlled trial (ImNuT). *BMC Pediatr.* 2021;21(1):19. | Trial protocol |
| 87 | Westerberg AC, Schei R, Henriksen C, et al. Attention among very low birth weight infants following early supplementation with docosahexaenoic and arachidonic acid. *Acta Paediatr*. 2011;100(1):47-52. | Wrong participants* |
| 88 | Willatts P, Forsyth S, Agostoni C, Casaer P, Riva E, Boehm G. Effects of long-chain PUFA supplementation in infant formula on cognitive function in later childhood. *Am J Clin Nutr*. 2013;98(2):536S-542S. | Wrong participants |
| 89 | Zhang J. Investigation of effects of supplementation of DHA-fortified milk powder during pregnancy and lactation on the health of mothers and infants: a randomized clinical trial. Chinese Clinical Trial Registry identifier: ChiCTR1800019461. Updated November 12, 2018. Accessed July 2, 2024. <https://www.chictr.org.cn/showprojEN.html?proj=32660> | Trial registration |

**Data not reported separately for infants < 29 weeks’ gestation*

**Supplementary Table 7. Risk of Bias of Included Trials**

| **RCT; citation(s) for follow up** | **Random sequence generation (selection bias)** | **Allocation concealment (selection bias)** | **Blinding of participants and personnel (performance bias)** | **Blinding of outcome assessment (detection bias)** | **Incomplete outcome data (attrition bias)** | **Selective reporting (reporting bias)** | **Notes** |
| --- | --- | --- | --- | --- | --- | --- | --- |
| DINO  Gould 2023 (18 months’ and 7 years’ CA) | Low risk: “computer-driven… randomization service according to an independently generated randomization schedule” | Low risk: “telephone randomization” | Low risk: “Parents, clinicians, and all research personnel were blinded to participant study group” and “All capsules were similar in size, shape, and color” | *18 months’ CA*  Low risk: “All study staff were blinded to treatment group allocation through the trial and follow-up assessments” | *18 months’ CA*  Low risk: 204/208 eligible children included (98%); missing outcome data balanced in numbers across groups, with similar reasons for  missing data across groups | Unclear risk:  No published study protocol, but study’s pre-specified outcomes reported as per trial registration (ACTRN12606000327583); however, this was a “post-hoc exploratory analysis… in infants born <29 weeks’ gestation” |  |
|  |  |  |  | *7 years’ CA*  Low risk: as above | *7 years’ CA*  Low risk: 196/205 eligible children included (96%); missing outcome data balanced in numbers across groups, with similar reasons for  missing data across groups |  |  |
| MOBYDIck  Guillot 2022 (18-22 months’ CA)  Paquet 2024  (5 years’ CA) | Low risk: “computer-generated randomization list” | Low risk: “The main trial pharmacist… coded the medication in advance according to randomization lists for each trial site” | Low risk: “Treatment bottles were identical and dispensed by site pharmacists blinded to the treatment allocation… All investigators, research staff, clinicians, pharmacists, the main trial statistician, and participants at each trial site and the data coordinating center were blinded from treatment allocation” | *18-22 months’ CA*  Low risk: “All participants, researchers, and clinicians responsible for the neurodevelopmental assessment were blinded to group allocation” | *18-22 months’ CA*  Low risk: 457/528 eligible children participated (87%); missing outcome data balanced in numbers across groups, with similar reasons for  missing data across groups | Low risk: study protocols published (18-22 months’ CA and 5 years’ CA) and pre-specified outcomes reported in pre-specified way (NCT02371460) | *18-22 months’ CA*  “There were no significant differences among the baseline characteristics of the children with and without data available at 18 to 22 months’ CA. However, mothers of children with follow-up data available at 18 to 22 months were older, with higher levels of education and a smaller proportion of them smoked during pregnancy in comparison with those without follow-up data available” |
|  |  |  |  | *5 years’ CA*  Low risk: “All participants, researchers and clinicians responsible for the neurodevelopmental assessment were blinded to the intervention.” | *5 years’ CA*  High risk: 132/177 eligible children participated (75%) |  |  |
| N3RO  Hewawasam 2021 (18 months’ and 2-3 years’ CA)  Gould 2023 (5 years’ CA) | Low risk: “A computer-generated randomization scheduled… was prepared by an independent statistician” | Low risk: “randomly assigned with the use of our Web-based randomization service” | Low risk: “Family members and clinical and research personnel were unaware of the feeding assignment until completion of the analyses” and “Emulsions were identical in viscosity, color, and packaging” | *18 months’ and 2-3 years’ CA*  Low risk: “clinicians and researchers remained blinded… until the completion of the attention assessments” and “Un-blinding of the follow-up study investigators only occurred after all analyses were completed” | *18 months’ CA*  High risk: 77/120 eligible children included (64%)  *2-3 years’ CA* Fewer (56, 47%) had BSID-III assessment; proportion of missing outcomes enough to have induced clinically relevant bias in observed effect estimates | Low risk: study protocols published (18 months’ and 2-3 years’ CA and 5 years’ CA) and pre-specified outcomes reported in pre-specified way (ACTRN12612000503820) | *18 months’ and 2-3 years’ CA*  “Baseline characteristics of children included in the follow-up study were somewhat different to the remainder of the N3RO cohort. Similarly, the baseline characteristics differed between eligible N3RO children who did and did not participate in the 18-month assessment” |
|  |  |  |  | *5 years’ CA*  Low risk: “Other trial staff and outcome assessors continued to be unaware of the group assignments throughout the 5-year follow-up” | *5 years’ CA*  Low risk:  WPPSI-IV: 480/656 (73%) eligible children participated; multiple imputation used for 176 (26%); missing data imputed using appropriate methods  SDQ and BRIEF: 731/958 (76%) eligible children participated; multiple imputation used for 227 (24%); missing data imputed using appropriate methods |  |  |

**Abbreviations:** ASQ: Ages and Stages Questionnaire; BRIEF: Behavior Rating Inventory of Executive Function; BSID-III: Bayley Scales of Infant and Toddler Development Third Edition; CA: corrected age; DINO: DHA for the Improvement of Neurodevelopmental Outcome in preterm infants; MOBYDIck: Maternal Omega-3 Supplementation to Reduce Bronchopulmonary Dysplasia in Very Preterm Infants; N3RO: N-3 Fatty Acids for Improvement in Respiratory Outcomes; RCT: randomised controlled trial; SDQ: Strengths and Difficulties Questionnaire; WPPSI-IV: Wechsler Preschool and Primary Scale of Intelligence Fourth Edition

**Supplemental Figures**

**Supplementary Figure 1. Risk of Bias of Included Trials**

**Abbreviations:** CA: corrected age; DINO: DHA for the Improvement of Neurodevelopmental Outcome in preterm infants; MOBYDIck: Maternal Omega-3 Supplementation to Reduce Bronchopulmonary Dysplasia in Very Preterm Infants; N3RO: N-3 Fatty Acids for Improvement in Respiratory Outcomes

**Supplementary Figure 2. Meta-analysis Results for Secondary Outcomes at 1-3 Years**

**A** Mild Cognitive Impairment: Bayley-II MDI or Bayley-III CC < 85

**B** Language Development Scores: Bayley-III LC

**C** Mild Language Impairment: Bayley-III LC < 85

**D** Moderate/Severe Language Impairment: Bayley-III LC < 70

**E** Motor Development Scores: Bayley-II PDI or Bayley-III MC

**F** Mild Motor Impairment: Bayley-II PDI or Bayley-II MC < 85

**Abbreviations:** Bayley-II: Bayley Scales of Infant and Toddler Development Second Edition; Bayley-III: Bayley Scales of Infant and Toddler Development Third Edition; CC: cognitive composite; CI: confidence interval; DHA: docosahexaenoic acid; DINO: DHA for the Improvement of Neurodevelopmental Outcome in preterm infants; IV: inverse variance; LC: language composite; MC: motor composite; MDI: mental development index; MOBYDIck: Maternal Omega-3 Supplementation to Reduce Bronchopulmonary Dysplasia in Very Preterm Infants; N3RO: N-3 Fatty Acids for Improvement in Respiratory Outcomes; PDI: psychomotor development index; SE: standard error

**Supplementary Figure 3. Meta-analysis Results for Secondary Outcomes at 4-8 Years**

**A** Verbal Scores: WPPSI-IV VCC or WASI VIQ

**B** Verbal Scores: WPPSI-IV VCC < 85 or WASI VIQ < 85

**C** Mild Cognitive Impairment: WPPSI-IV FSIQ or WASI FSIQ < 85

**D** SDQ Emotional Symptoms Score

**E** SDQ Conduct Problems Score

**F** SDQ Hyperactivity/Inattention Score

**G** SDQ Peer Relationship Problems Score

**H** SDQ Prosocial Behaviour Score

**I** SDQ Impact Score

**J** SDQ Total Difficulties Score

**K** BRIEF Inhibit Scale

**L** BRIEF Shift Scale

**M** BRIEF Emotional Control Scale

**N** BRIEF Working Memory Scale

**O** BRIEF Plan/Organise Scale

**P** BRIEF Flexibility Index

**Q** BRIEF Behavioural Regulation Index

**R** BRIEF Metacognition Index

**S** BRIEF Total Score: Global Executive Composite

Abbreviations: BRIEF: Behavior Rating Inventory of Executive Function; CI: confidence interval; DHA: docosahexaenoic acid; DINO: DHA for the Improvement of Neurodevelopmental Outcome in preterm infants; FSIQ; full-scale intelligence quotient; IV: inverse variance; MOBYDIck: Maternal Omega-3 Supplementation to Reduce Bronchopulmonary Dysplasia in Very Preterm Infants; N3RO: N-3 Fatty Acids for Improvement in Respiratory Outcomes; SDQ: Strengths and Difficulties Questionnaire; SE: standard error; WASI: Wechsler Abbreviated Scale of Intelligence; WPPSI-IV: Wechsler Preschool and Primary Scale of Intelligence Fourth Edition; VCC: verbal comprehension composite; VIQ: verbal intelligence quotient
